# Quinine Amaurosis, with Report of Three Cases

**Published:** 1910-08

**Authors:** B. H. Booth

**Affiliations:** Drew, Mississippi


For Texas Medical Journal.

### Quinine Amaurosis, With Report of Three Cases.

BY B. H. BOOTH, M. D., DREW, MISSISSIPPI.

Quinine amaurosis is occasionally met with in this section—the Yazoo and Mississippi valley, as a result of the continued or massive doses of quinine in severe cases of severe estivo-autumnal fever. It seems to be due to a temporary paralysis of the optic nerve, due most probably to the irritating properties of the drug in the blood. The eyes present no evidence of inflammation, such as heat, redness or swelling, there being little or no pain. The pupils are widely dilated, and, if the patient is undisturbed, he generally keeps the eyes closed. The general nervous system is distured, showing the systemic effect of the quinine, and there may be urticaria, tinnitis aurium, dizziness, etc. The treatment consists in stopping the drug, giving the patient tonics, etc., keeping the eyes protected from light and perfectly clean by the use of some mild antiseptic wash, such as saturated solution of boracic acid. The cases that I have seen have all recovered in from four to nine days, with apparently normal vision.

CASE 1.—P. S., Hebrew, male, age 9 years, developed a severe case of estivo-autumnal malaria; received usual treatment, "cleaning out," and then for three days thirty grains of quinine sulphate per day. On morning of fourth day temperature reached normal, and quinine was stopped at 6 a. m. About 9 he complained of not being able to see well and was totally blind by 12 m. He received the treatment outlined above and recovered complete sight on the fourth day afterwards.

Case 2.—M. S., negro, female, age 3 years, had a severe case of estivo-autumnal fever, algid form; received usual treatment for same, consisting of calomel and soda to cleanse out the bowels and promote the secretions, hot baths, etc., and thirty grains of quinine and urea hydrochloride hypodermatically repeated in twelve hours. This was all the quinine exhibited, as the patient responded beautifully, but about forty-eight hours later on awakening from sleep she was unable to see at all. Same treatment as in the preceding case, which resulted in gradual return of vision which was completed in about one week.

Case 3.—I saw this case after amaurosis was complete. White, male, age about 40, had been in bed with estivo-autumnal fever for about two weeks, and had been treating himself. For the past ten days had been taking five grains of quinine sulphate every four hours. I stopped same and treated him same as two preceding cases, with complete recovery of vision in about eight days.

In an extensive practice of eight years in a highly malarious locality, where enormous doses of quinine are constantly being exhibited, I have seen only these three cases, but have heard of several more in the community. It will be noticed that these cases are all of different races, while two are in children and one in an adult, also that two are males and one female. While the exciting cause is undoubtedly quinine, I am inclined to believe that the effect of the malaria on the system of the patient is a contributing factor. These cases are reported because I am not able to find much on this condition in the literature.

